# RNA sequencing data analysis of the yeast *Vanrija* (*Cryptococcus*) *humicola* strain UJ1 grown on l- and d-aspartate

**DOI:** 10.1016/j.dib.2023.109008

**Published:** 2023-02-23

**Authors:** Daiki Imanishi, Shouji Takahashi

**Affiliations:** Department of Material Science and Bioengineering, Nagaoka University of Technology, Nagaoka, Niigata 940–2188, Japan

**Keywords:** Yeast, *Vanrija humicola*, RNA-seq, d- and l-Aspartate, d-amino acid

## Abstract

The yeast *Vanrija* (previously *Cryptococcus*) *humicola* strain UJ1 produces d-aspartate oxidase (DDO) only in the presence of d-aspartate in culture media. This article provides RNA-sequencing data to identify the differentially expressed genes (DEGs) in the yeast cells grown between l- and d-aspartate. RNA samples were prepared from the yeast cells grown in a culture medium containing 30 mM d-aspartate or l-aspartate as the sole carbon source and subjected to RNA sequencing on Illumina NovaSeq6000 platform. The clean reads obtained by removing adaptor sequences and low-quality reads from raw reads were submitted to the Sequence Read Archive (SRA) database of the National Center for Biotechnology Information (NCBI) under the BioProject accession number PRJDB13570. The clean reads were subjected to differential gene expression analysis using DEGSeq to provide data on the upregulated and downregulated DEGs in the cells grown on d-aspartate. The DEGs were subjected to gene ontology (GO) and KEGG pathway enrichment analyses using GOSeq and KOBAS, respectively, to provide data on the possible biological functions of the DEGs. The data set obtained in this project might be helpful for further investigation of the effects of d-aspartate on cellular processes in yeast cells and other eukaryotic organisms.

**Specifications Table**

**Table:** 

Subject	Biological science: Microbiology: Fungal Biology
Specific subject area	Differentially expressed genes in the yeast *V. humicola* strain UJ1 grown on l-aspartate vs. d-aspartate as the sole carbon source
Type of data	Clean, Tables, Graphs
How the data were acquired	RNA Sequencing on Illumina NovaSeq 6000 PE150 platform Software: TopHat v2.0.12, HTseq v0.6.1, DEGSeq v1.12.0, GOSeq package (GOSeq, topGo, hmmscan) release 2.12, KOBAS v3.0.
Data format	Clean sequence data: Illumina FASTQ files Analyzed data: Tables, Graphs
Description of data collection	Cells were incubated for 5 h at 30°C in a medium containing 30 mM d-aspartate or l-aspartate as the sole carbon source, and total RNA was extracted. After mRNA enrichment by rRNA depletion and oligo dT selection, RNA was randomly fragmented, followed by cDNA synthesis using random hexamers. The second strand was generated by nick-translation. The synthesized cDNA was subjected to end-repair and then was 3′ adenylated. Sequencing adaptors were ligated to the ends, and the final products were size selected and enriched by PCR, followed by sequencing on the Illumina NovaSeq 6000 PE150 platform.
Data source location	Department of Material Science and Bioengineering, Nagaoka University of Technology City/Town/Region: Kamitomioka, Nagaoka, Niigata Country: Japan
Data accessibility	FASTQ file of *V. humicola* strain UJ1 grown on l-aspartate and d-aspartate can be found at Sequence Read Archive on the NCBI database website with the accession numbers DRR395702 and DRR395703, respectively, under BioProject accession number PRJDB13570. Direct URL to data: https://www.ncbi.nlm.nih.gov/sra/?term=PRJDB13570 The supplemental files are provided as Microsoft Excel (.xlsx) and can be found in Mendeley repository data. Direct URL to data: https://data.mendeley.com/datasets/vhsy73dryt/1 DOI:10.17632/vhsy73dryt.1

## Value of the Data

• This data set provides insights into the effect of d-aspartate on the gene expressions in yeast cells and could also provide insights into the effect of d-aspartate on other eukaryotic cells.
• The gene expression data provide a comprehensive picture of transcriptomic change affected by d-aspartate in the yeast cells and other eukaryotic cells.
• These data are valuable resources for scientific communities working on d-amino acids in organisms to understand the molecular functions and processes affected by d-amino acids.

## 1 Objective

Recently, free d-amino acids have been found in various organisms, including humans, and shown to play important roles in various physiological processes [1]. In the human brain, they are involved in neurotransmission and hormone synthesis and secretion [2]. In eukaryotic organisms, endogenous and exogenous d-aspartate is metabolized mainly by DDO [3,4]. In the yeast *V. humicola* strain UJ1, DDO (ChDDO) plays a role in the utilization of d-aspartate for cell growth and functions as a detoxifying enzyme for d-aspartate toxicity [5]. DDO in the yeast is transcriptionally induced in response to the presence of d-aspartate in cultivation media, indicating that there might be a regulation mechanism of d-aspartate-dependent gene expression. Interestingly, similar induction of gene expression and enzyme activity in response to the presence of d-amino acids has been observed in various eukaryotic organisms, including mammals [6], [7], [8], [9]. However, the mechanism of d-amino acid-dependent gene expression has not been fully elucidated. In addition, the effects of d-amino acids, including d-aspartate, on whole-cellular processes and functions in eukaryotic organisms are largely unknown. Therefore, we performed an RNA-seq analysis of the yeast *V. humicola* strain UJ1 cells cultivated on d-aspartate or l-aspartate as the sole carbon source.

## 2 Data Description

**Fig. 1:**
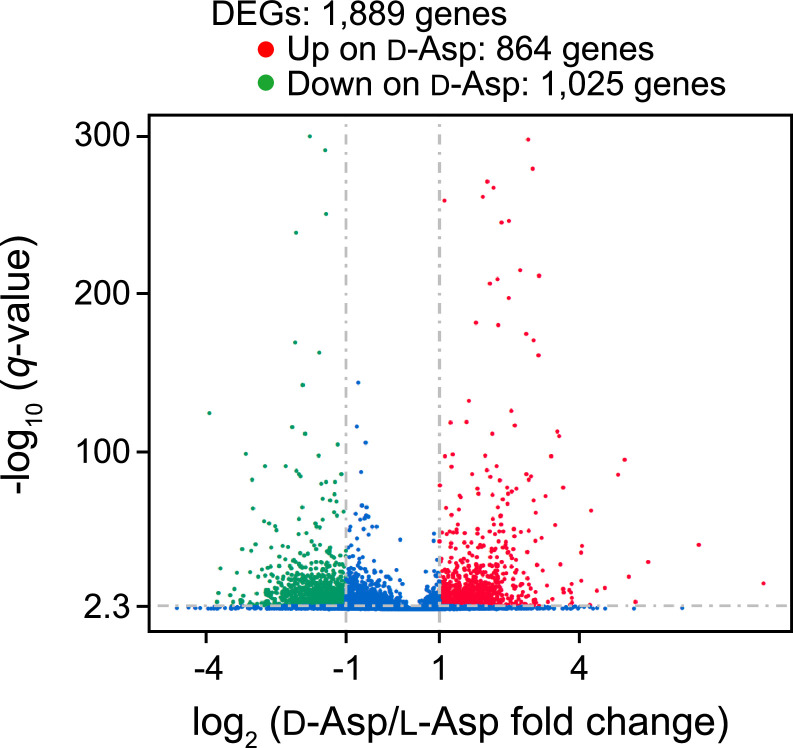
Volcano plot of the RNA-seq analysis of the yeast *V. humicola* strain UJ1 cells grown on l- and d-aspartate as the sole carbon source. The upregulated and downregulated DEGs (absolute log_2_ fold change > 1 and *q*-value <0.005, supplemental file 1) in the cells grown on d-aspartate compared with l-aspartate are represented in red and green dots, respectively.

**Fig. 2:**
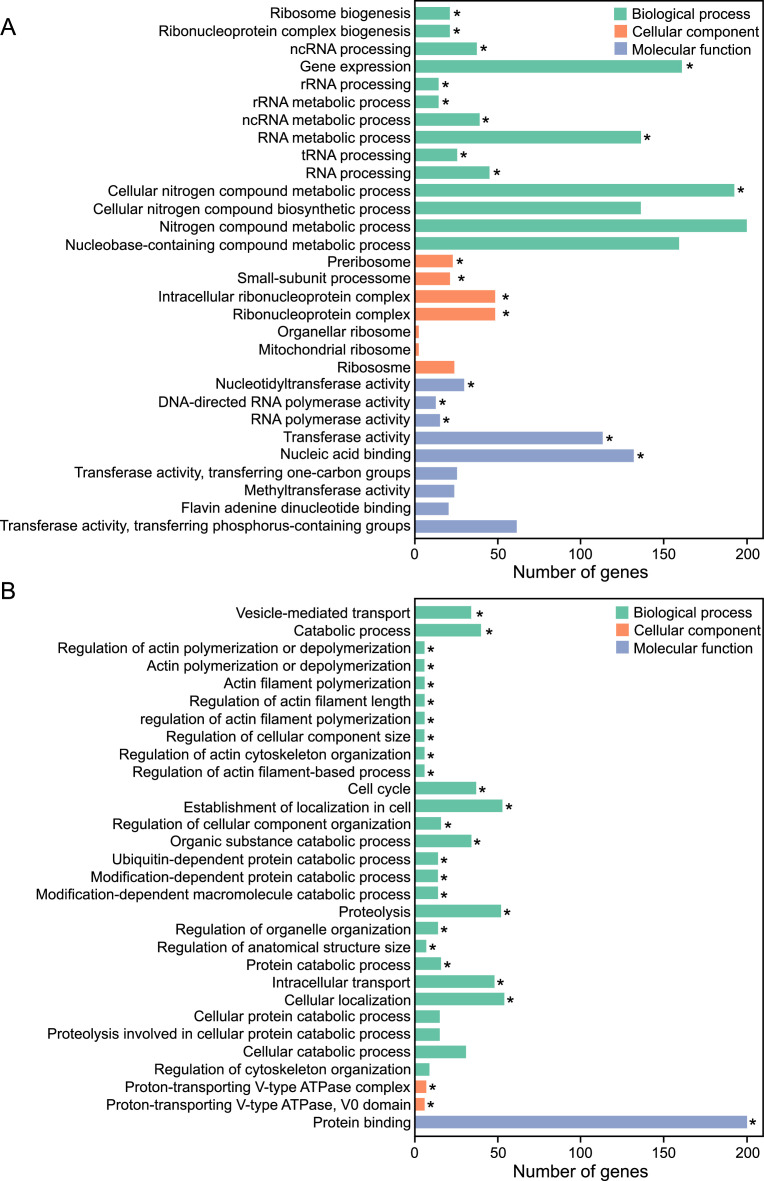
GO enrichment analysis of the upregulated (A) and downregulated (B) DEGs in the yeast *V. humicola* strain UJ1 cells grown on d-aspartate as the sole carbon source in comparison with l-aspartate. The top 30 Go terms enriched in upregulated and downregulated DEGs are classified into three categories (supplemental files 2 and 3, respectively), Biological process, Cellular component, and Molecular function, and shown in green, orange, and blue, respectively. The asterisks indicate statistically significant differences (*q* < 0.05).

**Fig. 3:**
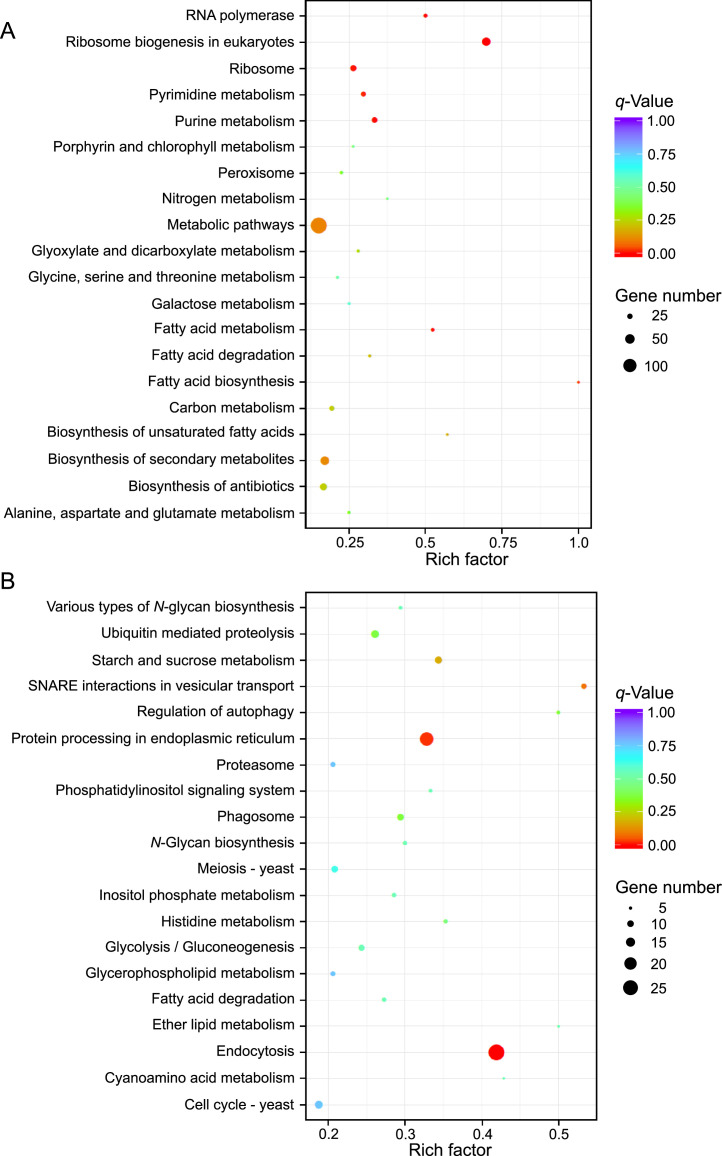
KEGG pathway enrichment analysis of the upregulated (A) and downregulated (B) DEGs in the yeast *V. humicola* strain UJ1 cells grown on d-aspartate as the sole carbon source in comparison with l-aspartate. The top 20 KEGG pathways enriched in upregulated and downregulated DEGs are represented by the number of genes enriched to each pathway (supplemental files 4 and 5, respectively). Rich factor represents a number of all annotated genes under each pathway term. The color of the dots represents transitional values expressed as -log_10_ of *q*-value, and the size indicates the number of genes enriched to each term.

This article reports the RNA sequencing data of the yeast *V. humicola* strain UJ1 grown on l-aspartate or d-aspartate as the sole carbon source. Table 1 shows the descriptive data statistics of raw and clean RNA-seq reads. The clean RNA-seq reads were deposited in the DDBJ Sequence Read Archive (SRA) repository (https://www.ncbi.nlm.nih.gov/sra/?term=PRJDB13570, BioProject ID PRJDB13570) under the accession numbers DRR395702 for l-aspartate and DRR395703 for d-aspartate. Fig. 1 shows a volcano plot of differentially expressed genes (DEGs, absolute log_2_ fold change > 1 and *q*-value <0.005) between the yeast cells grown on l-aspartate and d-aspartate, and “Supplemental File1” (https://data.mendeley.com/datasets/vhsy73dryt/1) shows the details: the gene id, read counts, log_2_ fold change, *p*-value, *q*-value, and DEG signature. Fig. 2 and 3 show top 30 gene ontology (GO) terms and top 20 KEGG pathways, respectively, enriched in upregulated and downregulated DEGs in the cells grown on d-aspartate in comparison with l-aspartate. “Supplemental Files 2 and 3” (https://data.mendeley.com/datasets/vhsy73dryt/1) provide the details of the upregulated and downregulated DEGs, respectively, in the GO enrichment analysis: GO accession (Gene Ontology entry), description (detail description of Gene Ontology), term type (GO types, including cellular component, biological process, and molecular function), over-represented *p*-value (*p*-value in hypergeometric test), corrected *p*-value (GO with corrected *p*-value < 0.05 are significantly enriched in DEGs), DEG item (number of DEGs with GO annotation), DEG list (number of all reference genes with GO annotation), Bg item (number of background genes related to this GO), Bg list (number of all the background genes with GO annotation), and gene ID (ID of DEGs related to this GO. “Supplemental Files 4 and 5” (https://data.mendeley.com/datasets/vhsy73dryt/1) provide the details of the upregulated and downregulated DEGs, respectively, in the KEGG pathway enrichment analysis: Term (the description of KEGG pathways), KEGG ID (reference gene IDs of *Cryptococcus neoformans* var. *neoformans* strain JEC21), input number (number of DEGs with pathway annotation), background number (number of all reference genes with pathway annotation), *p*-value (*p*-value in hypergeometric test), corrected *p*-value (KEGG pathway with corrected *p*-value < 0.05 are significantly enriched in DEGs), input gene ID (ID of DEGs related to the Pathway), KEGG ID/KO (the KEGG ID or KO number of DEGs enriched), Entrez ID (the NCBI-Gene ID of DEGs enriched), and hyperlink URL (direct address to the position of DEGs in the KEGG pathway of *C. neoformans* var. *neoformans* strain JEC21 as a reference).

**Table 1:** The RNA-seq descriptive statics data of the yeast *V. humicola* strain UJ1 grown on l- and d-aspartate as the sole carbon source

	Value	
Descriptive	*V. humicola* strain UJ1 grown on l-aspartate	*V. humicola* strain UJ1 grown on d-aspartate
Total number of raw reads (bp)	49,934,972	44,003,866
Total number of raw bases (Gb)	7.5	6.6
Total number of clean reads (bp)	48,813,266	42,953,666
Total number of clean bases (Gb)	7.3	6.4
Clean Reads Q30 (%)	93.61	93.67
GC Content (%)	63.65	63.64
Biosample ID	SAMD00510952	SAMD00510953
DRR number	DRR395702	DRR395703

Total number of clean reads: number of reads after filtering of raw reads.

Total number of clean bases: clean reads number multiply read length, saved in G unit.

Clean Reads Q30 (%): percentages of bases whose correct base recognition rates are greater than 99.9% in total bases.

GC content (%): percentages of G and C in total bases.

## 3 Experimental Design, Materials and Methods

### 3.1 Cultivation

*V. humicola* strain UJ1 cells were cultivated in SD medium (0.67% yeast nitrogen base w/o amino acids with ammonium sulfate, 2% glucose) at 30°C for 16 h. Then, an aliquot of the culture was transferred to 100 mL of fresh SD medium with a final OD_600_ of 0.05 and cultivated at 30°C for 16 h. The cells were collected by centrifugation at 5,000 × g for 10 min at 4°C and washed twice with ice-cold sterilized water. The cells were suspended in YNB medium (0.67% yeast nitrogen base w/o amino acids with ammonium sulfate) containing 30 mM NH_4_Cl and 30 mM d-aspartate or l-aspartate to an OD_600_ unit of 10 and cultivated at 30°C for 5 h. The cells were collected by centrifugation at 5,000 × g for 10 min at 4°C, washed twice with ice-cold sterilized water, and transferred to a 2.0 mL screw-cap tube containing 0.2 g of zirconia beads (0.45−0.5 mm in diameter). After removing the supernatant, the cells were lyophilized using a freeze dryer (DRC-1100, EYELA, Tokyo, Japan) and kept at -80°C until use.

### 3.2 Extraction and sequencing of RNA

The lyophilized cells in a screw-cap tube containing zirconia beads were vortexed vigorously for 5 min. Total RNAs were extracted from the cell homogenate using the Direct-zol RNA Miniprep Kit (ZYMO Research, Irvine, CA, USA) according to the instructions of the manufacturer. The quality of total RNA was evaluated using NanoDrop Spectrophotometer (OD_260_/OD_280_) (NanoDrop Technologies Inc., Wilmington, DE, USA), agarose gel electrophoresis, and Agilent 2100 Bioanalyzer (Agilent Technologies, Palo Alto, CA, USA). After the quality check, mRNA was enriched using oligo(dT) beads and then fragmented randomly in a fragmentation buffer, followed by cDNA synthesis using random hexamers and reverse transcriptase. After first-strand synthesis, a custom second-strand synthesis buffer (Illumina, San Diego, CA, USA) was added with dNTPs, RNase H, and *Escherichia coli* polymerase I to generate the second strand by nick-translation. The final cDNA library is ready after a round of purification, terminal repair, A-tailing, ligation of sequencing adapters, size selection, and PCR enrichment. The library concentration was first quantified using a Qubit 2.0 fluorometer (Life Technologies, Carlsbad, CA, USA), then diluted to 1 ng/µl before checking insert size on an Agilent 2100, and quantifying to greater accuracy by quantitative PCR (library activity >2 nM). The constructed cDNA library was sequenced using the IlluminaNova Seq 6000 PE150 platform.

### 3.3 Bioinformatic analyses

Image analysis and base calling were performed using the Illumina CASAVA pipeline v1.8. The raw reads were filtered to remove the reads containing adapter contamination and the reads in which uncertain nucleotides constitute more than 10% of either end and low-quality nucleotides (base quality less than 20) constitute more than 50% of the reads. All clean reads were then aligned to the genome sequence of the yeast *V. humicola* strain UJ1 (DDBJ/EMBL/GenBank accession numbers BFAH01000001 to BFAH01000046; scaffolds 1 to 19 and 21 to 47, respectively) using the TopHat2 package [10]. Transcript abundance was quantified using HTSeq package v0.6.1 [11], and differentially expressed genes (DEGs) between the growth conditions on l- and d-aspartate were identified using the DEGSeq v1.12.0 [12]. The Benjamini–Hochberg false discovery rate (FDR) multiple test correction was applied, and genes with a log_2_ fold change > 1 or < -1 and with FDR adjusted *p*-value (*q*-value) <0.005 were considered to be differentially expressed. Gene Ontology (GO) enrichment analysis was performed using GOSeq package release 2.12 [13]. Kyoto Encyclopedia of Genes and Genomes (KEGG) enrichment analysis was performed using KOBAS v3.0 to annotate the function of DEGs and map into the KEGG pathway of the yeast *C. neoformans* var. *neoformans* strain JEC21 as a reference [14].

## Ethics Statements

This work does not involve any type of human studies, animal studies, or data gathered using social media.

This manuscript adheres to ethics in publishing standards.

## Supplementary Materials

Supplementary materials associated with this article can be found, in the online version, at doi:10.17632/vhsy73dryt.1 (Original data) (Mendeley Data).

## CRediT authorship contribution statement

**Daiki Imanishi:** Visualization, Methodology, Formal analysis, Data curation, Writing – original draft. **Shouji Takahashi:** Conceptualization, Visualization, Formal analysis, Data curation, Supervision, Writing – review & editing, Project administration.

## Declaration of Competing Interest

The authors declare that they have no known competing financial interests or personal relationships that could have appeared to influence the work reported in this paper.

## Data Availability

RNA sequencing data set of the yeast Vanrija humicola strain UJ1 grown on L- and D-aspartate (Original data) (DDBJ).
